# A clinical research priority setting study for issues related to the use of methamphetamine and emerging drugs of concern in Australia

**DOI:** 10.1111/dar.13350

**Published:** 2021-07-08

**Authors:** Krista J. Siefried, Nadine Ezard, Michael Christmass, Paul Haber, Robert Ali, The NCCRED Methamphetamine and Emerging Drugs Clinical Research Network Working Group

**Affiliations:** ^1^ The National Centre for Clinical Research on Emerging Drugs Sydney Australia; ^2^ Alcohol and Drug Service St Vincent's Hospital Sydney Sydney Australia; ^3^ The National Drug and Alcohol Research Centre UNSW Sydney Sydney Australia; ^4^ Next Step Drug and Alcohol Services Mental Health Commission Perth Australia; ^5^ National Drug Research Institute Curtin University Perth Australia; ^6^ Faculty of Medicine and Health Central Clinical School, Discipline of Addiction Medicine University of Sydney Sydney Australia; ^7^ Drug Health Services Royal Prince Alfred Hospital Sydney Australia; ^8^ School of Medicine Adelaide University Adelaide Australia

**Keywords:** research priority, substance‐related disorder, methamphetamine, health policy, drug user

## Abstract

**Introduction:**

This study aimed to gather a range of opinions, including those of affected people (consumers, concerned others) to identify clinical research priorities for methamphetamine and emerging drugs of concern in Australia, to guide the work of the National Centre for Clinical Research on Emerging Drugs (NCCRED).

**Methods:**

A priority setting study was conducted (February–March 2019) in four phases: online stakeholder survey, thematic analysis of responses, rapid literature review, expert panel ranking of priorities against predetermined criteria.

**Results:**

Forty‐seven respondents completed the survey, including people identifying as one or more of: researcher (53%, *n* = 25), clinician (45%; *n* = 21), family/friend/caregiver of someone who uses methamphetamine/emerging drugs (15%, *n* = 7) and consumer of methamphetamine/emerging drugs (13%, n = 6). Expert panel, evidence‐informed top‐ranked clinical research priorities for methamphetamine were: strategies to overcome barriers to intervention uptake, pilot medication trials for adults seeking treatment, and communication strategies regarding evidence‐based treatments. For emerging drugs of concern, top‐ranked priorities were: piloting community‐located drug checking, feasibility of social media/other opportunities to alert consumers of emerging risks, GHB overdose and withdrawal management, and impacts of an early warning information system on reducing harms.

**Discussion and Conclusions:**

We demonstrate feasibility of a structured, collaborative clinical research priority setting process. Results have informed the establishment of NCCRED; using the identified priorities to guide seed funding, fellowships/scholarships and research programs. Broader uptake of this methodology by policymakers/research funders would assist to embed areas of concern identified by affected communities and other stakeholders in research prioritisation.

## Introduction

Methamphetamines and other emerging drugs of concern are global issues with public health implications affecting individuals, families, workplaces and communities [1]. The Australian National Drug Strategy Household Survey (NDSHS) reported that in Australia, 1.3 million people over the age of 14 years (approximately 6.3% of this population) reported any lifetime use of methamphetamine(s), while 1.4% reported consumption in the past 12‐months [2]. In response to community concerns about crystal methamphetamine (‘ice’), the Australian government established a ‘National Ice Action Strategy’ [3]. The National Centre for Clinical Research on Emerging Drugs (NCCRED) was funded through the National Ice Action Strategy, to build clinical research capacity and work towards establishing evidence‐based treatment approaches for both methamphetamine(s) and emerging drugs of concern.

In establishing NCCRED, the Australian government adopted a broad definition of emerging drugs of concern to the Australian population, highlighting novel psychoactive substances (NPS) (e.g. synthetic cannabinoids, acetyl‐fentanyl) [4]. Formulations of older drugs/classes for which problems related to their use in Australia are emerging (e.g. fentanyl) were also included. The NDSHS began collecting NPS data in 2016, most recently reporting 0.7% of respondents had consumed an NPS [5]. Definitions and example drugs provided to respondents changed in subsequent surveys, and drugs, such as gamma hydroxybutyrate (GHB) and ketamine, are evaluated separately [5], making trends difficult to interpret. However, in the 2020 national ecstasy and related drugs reporting system, nearly a quarter of participants reported consuming NPS in the prior 6 months [6]. Despite a low prevalence of GHB use in the NDSHS (1% reported lifetime use [5]), gay and bisexual men report using GHB at a rate nearly 20 times higher [7], and between 2012 and 2019 there was a 147% increase in GHB‐related ambulance call‐outs in the Australian state of Victoria [8].

To focus the Centre's research and program objectives, NCCRED opted to undertake a clinical research priority setting process. Broadly, health research priority setting aims to select priorities that will have the largest benefit to the health of populations, reduce the duplication of research efforts and promote collaboration [9]. There is an obligation to effectively allocate resources to areas of greatest burden and answer research questions that have the capacity to produce the greatest impact. Research priority setting processes are frequently used at organisational, jurisdictional, national and international levels and there has been a shift towards more transparent and reproducible methods [10].

Several processes are identified in the literature for setting research priorities (Table 1). These include engaging with or consulting various stakeholders to provide contributions towards determining priorities. Depending on the method, stakeholders may include: patients, communities, caregivers, researchers, policymakers, health‐care providers, experts, institutions/organisations and individuals outside the health sector. In the case of consumer stakeholders (e.g. patients), some methods consider inclusion to be imperative [13, 17, 18, 19, 21], while they are excluded from others [11, 12, 15, 16]. There are growing calls to involve consumers in research priority setting broadly [23], and in the context of drug and alcohol research [24].

**Table 1:** Methods for health research priority setting

Method	Development	Brief description
CHNRI Process [11, 12]	CHNRI of the Global Forum for Health Research (WHO)	A systematic algorithm for decision on priorities. Experts are identified. Research ideas are generated by the experts or a management team based on the current evidence. Experts are requested to provide three research ideas against a pre‐determined domain of health research; generally via an electronic survey with results compiled by the management team. Scoring criteria include: answerability, equity, impact on burden, deliverability, effectiveness. Each criteria is scored on a scale (i.e. 0, 0.5 and 1, or 0–100). Pooling of individual scoring of research options based on the criteria.
CAM [13]	Global Forum for Health Research (WHO)	Focus on the structured collection of information. A structured framework for collecting information adhering to criteria for research priority setting and accounting for influence of different participants and factors. Institutional approach involving individuals, household and community; health ministry; health institutions; other sectors outside of health; macroeconomic level actors. Five‐step process: measure the disease burden, analyse determinants, evaluate present knowledge status, evaluate cost and effectiveness, present resource flows. Workshops and brainstorming. Each body feeds into a matrix (about a specific disease or factor) and the matrix identifies the level of information which then determines the candidates for research. Topics are grouped and cut‐down to prioritise areas.
Council on Health Research for Development's Approach [14]	Council on Health Research for Development	Management process for national approach, utilises other methods (e.g. ENHR, CAM, Delphi) to identify priority issues, and allows for using multiple methods. Ranking techniques are used to score.
Delphi Process [15, 16]	The United States Air Force Project RAND (The RAND Corporation)	Panel of experts and questionnaires. Suggest that groups consist of: top management/decision makers who will utilise the results; professional staff members and their support teams; respondents to the questionnaires. Round 1: open‐ended questionnaire sent to pre‐determined content area. Investigators turn this into a structured survey for data collection. Rounds 2–4: experts answer surveys, anonymously summarised by a facilitator, experts revise their earlier responses. Up to four rounds can be used.
ENHR Method [17, 18, 19]	Commission on Health Research for Development	Broad‐based consultation with communities, researchers, policymakers, health‐care providers. Stakeholders suggest priority areas using evidence‐based situation analysis. Criteria include appropriateness, relevancy, likelihood for success, and potential impact. Scores are assigned. Consensus is achieved through brainstorming, multi‐voting, nominal group technique, round table discussions.
JLA Method [20]	The JLA is a non‐profit initiative developed in 2004, managed and coordinated by the National Institutes for Health Research Evaluation, Trials and Studies Coordinating Centre at the University of Southampton	Equal partnership of patients, caregivers and clinicians to agree through consensus. Treatment uncertainties are identified and confirmed by systematic reviews of databases to verify research gaps. This requires a confidence interval in a systematic review to not cross the line of effect. Consensus agreement (no scoring criteria clearly defined in the method) to prioritise a ‘top 10’ list of the uncertainties. Public availability of the methods and results of the priority setting partnership. Bring the results of the process to the research funders (independent of the JLA).
Nine Common Themes of Good Practice [21]	Based on literature and WHO, to determine the common themes of good practice of health research priority setting exercises	A checklist for developing health research priority setting exercises; to allow informed choice on approaches and provision of generic assistance to plan research priority setting based on nine common themes of good practice. These are: context, comprehensiveness of approach, inclusiveness, information gathering, planning for implementation, criteria, methods for deciding on priorities, evaluation, transparency.
Sibbald's Conceptual Framework for successful priority setting [22]	A 2009 Canadian publication synthesizing other work to develop a conceptual framework for research priority setting	Review of three empirical studies (reporting use of: Delphi method, one‐on‐one phone interviews based on literature, or focus groups) to provide a framework of 10 separate and interconnected elements key to effective priority setting. The elements of successful priority setting (for process and outcome) were identified as: stakeholder understanding, shifted priorities/reallocation of resources, decision‐making quality, stakeholder acceptance and satisfaction, positive externalities, stakeholder engagement, use of explicit process, information management, consideration of values and context and revision or appeals mechanism.

CAM, Combined Approach Matrix; CHNRI, Child Health and Nutrition Research Initiative; ENHR, Essential National Health Research; JLA, James Lind Alliance; WHO, World Health Organization.

There is no ‘gold standard’ method for health research priority setting [21, 22], leading some authors to assert that any method is preferable to none [10]. In developing the NCCRED approach, we drew on the conceptual framework described in the ‘Nine Common Themes of Good Practice’ checklist [21]. The purpose of the checklist is to facilitate the planning and implementation of a research priority setting process, and it has also been applied as an evaluation tool [25]. The checklist identifies nine key elements and recommends these be included in any research priority setting exercise [21].

Research priorities can be set at a broad level (e.g. a region or therapeutic area), intermediate level (e.g. shared by a sector) or at the level of specific research questions [26]. NCCRED was funded by the Commonwealth Department of Health to address methamphetamine and emerging drugs of concern (at the broad level), so the aim of this research priority setting study was to identify intermediate and specific level priorities, with a focus on clinical research. Embedding consumer, family/friend/caregiver and community priorities in the research effort is a key aim of NCCRED, and in line with increasing calls for inclusion of affected populations in setting the research agenda [24, 27]. We therefore considered the opinions of affected communities when designing our approach. This report aims to: (i) outline the methods undertaken in the NCCRED clinical research priority setting study to drive the work of the Centre; and (ii) report the results, including limitations of the NCCRED clinical research priority setting study.

## Methods

The purpose of the checklist in the ‘Nine Common Themes of Good Practice’ is to facilitate planning and implementation of a research priority setting process [21]. The checklist identifies nine key elements and recommends their inclusion in any research priority setting exercise [21]. These are: context, comprehensiveness of approach, inclusiveness, information gathering, planning for implementation, criteria, methods for deciding on priorities, evaluation and transparency. There are overlapping themes in the methods previously described and therefore several components of other methodologies were incorporated, or were also satisfied by, the methods we used (see Table 2).

**Table 2:** Evidence underpinning process

Nine Common Themes of Good Practice [21]^a^	NCCRED approach and Phase of Study	Other methods satisfied by this approach
**Preparation**		
* Context* Describe the contextual factors underpinning the process	NCCRED established as part of the National Ice Action Strategy [3], following recommendations made by the National Ice Taskforce [28]. Funding provided to NCCRED includes allocated resources to develop two NCCRED‐sponsored clinical trials as well as developing an NCCRED funding program to provide seed‐funding for clinical research, and to establish a clinical research fellowship program Scope of the priority setting study: identification of the priority clinical research areas that can deliver results within the NCCRED timeframe and budget, and also, in the broader Australian context, to better inform policymakers, academics, clinicians, etc. The intended clinical research beneficiaries are people who are impacted by methamphetamine and emerging drug use	CAM [13]
* Use of comprehensive approach* Assess whether a comprehensive approach or tailored process is required	A structured, detailed and step‐by‐step guide to the priority setting process that NCCRED will engage in. The study will involve a survey followed by a tailored process of an independent panel of expert reviewers to synthesise the results of the survey and provide recommendations based on consensus	CAM [13] CHNRI [11, 29] COHRED [14] ENHR [17, 18, 19] Sibbald [22] Delphi [15, 16]
* Inclusiveness* Determine the stakeholders who should be involved in setting research priorities	Elected to aim for broad stakeholder involvement, to minimise the chances of research options being overlooked, foster a sense of ownership of the established priorities among those involved, and increase the potential for implementation of the priorities Aimed for this study to broadly reflect the needs of those implementing the research as well as those who will potentially benefit from the research. Broad stakeholder engagement enables the study results to potentially increase the impact on health and health equity	CAM [13] COHRED [14] ENHR [17, 18, 19] JLA [20] Sibbald [10, 22]
* Information gathering* Select what information should be gathered to inform the process	Information gathered from a survey, including structured and open‐ended questions available to all stakeholders, brief literature review following assessment of themes reported by the survey, expert panel to provide information with survey and review information available	
* Planning for implementation* Establish plans for translation of research priorities into projects	The independent expert panel review of respondents' feedback aimed to independently inform NCCRED on the areas of focus for NCCRED programs to be supported (direct translation of priorities into funded projects)	
**Deciding on priorities**		
* Criteria* Select relevant criteria to focus discussion around research priorities	To focus the discussion of the survey responses by the independent expert panel a set of criteria were used, broadly based around the themes of: public health benefit; feasibility; and cost. The following were considered (informed by NCCRED aims and the priority setting processes previously discussed): Able to deliver results within 2 years Assists with building the AOD sector's research capacity Able to involve multiple sites nationally (equity), including rural and remote areas Focused on clinical treatment of methamphetamine use disorder and/or emerging drugs of concern (appropriateness) Can be sponsored by NCCRED Evidence can be translated into clinical practice (potential impact) Can be developed into research questions that will produce findings in order to change treatment outcomes Impact is of population significance Can be developed into research questions that promote opportunities for collaboration and partnerships Ability to develop current areas of research practice or excellence to build capacity (builds on existing structures, research strengths, data sources—relevancy) There is a gap in the current evidence (impact on burden, relevancy, appropriate) Answerability / likelihood for success	CAM [13] CHNRI [11, 29] COHRED [14] ENHR [17, 18, 19]
* Methods for deciding on priorities* Choose a method for deciding on priorities	Results of the survey were analysed first thematically, and then by an independent panel of experts. The independent expert panel used a combination of scoring and consensus. This allowed for the diversity of experience within the expert panel and aimed to improve the acceptability of the process and its results.	CAM [13] CHNRI [11, 29]
**After priorities set**		
* Evaluation* Define when and how evaluation of process and outcome will occur	A brief impact analysis conducted by NCCRED as part of the analysis and manuscript preparation. In addition, limitations and strengths of the process and lessons learned were examined and reported to ensure that future, similar exercises are able to benefit from the learnings in this project	
* Transparency* Communicate the approach used to set priorities	Following the study, results submitted for peer‐reviewed publication, and reported in the grey literature [30]	JLA [20]

^a^ *Description* as reported in the Nine Common Themes Checklist [21]. AOD, alcohol and other drugs; CAM, Combined Approach Matrix; CHNRI, Child Health and Nutrition Research Initiative; COHRED, Council on Health Research for Development; ENHR, Essential National Health Research; JLA, James Lind Alliance; NCCRED, National Centre for Clinical Research on Emerging Drugs.

The study consisted of four phases: (i) an online survey of stakeholders (described below); (ii) a qualitative thematic analysis (assessment of survey responses); (iii) a literature review assessing the themes identified by respondents against published peer‐reviewed data; and (iv) results and literature review were presented to an expert panel and through discussion, consensus and ranking, the clinical research priorities were determined. The expert panel was independent of NCCRED's Board, the funding allocated to NCCRED and NCCRED staff.

The study survey aimed to engage with stakeholders nationally, at all levels of clinical, service, and research delivery and utilisation, including: consumers, concerned others (e.g. family, friends)/caregivers, clinicians, researchers, policymakers, industry, research funders, institutions/organisations, law enforcement, border control and other interested community members. A newsletter with a link to the online survey was emailed to the NCCRED mailing list and links to the survey were promoted and disseminated through NCCRED supporters/partners via organisational newsletters, Twitter feeds and other networks. Links to the survey were distributed to professional interest groups, medical chapters/colleges, consumer advocacy groups, government and non‐governmental organisations. Recipients were invited to forward the email to other interested parties. The link was also available through the NCCRED website.

The online survey was accessed after the participant completed an online informed consent and acknowledged their consent to participate. Ethics approval was obtained from the St Vincent's Hospital Human Research Ethics Committee (2018/ETH00671). There was no reimbursement for participation.

The survey asked participants to list areas in the treatment and management of methamphetamine use and emerging drugs of concern that they felt would benefit from clinical research. The Australian Government National Health and Medical Research Council definition of clinical research was provided as follows: ‘Clinical research increasingly involves a range of different health professionals studying a wide range of matters, including disease prevention and causation, diagnostic methods, treatments, and effects of and response to illness. Such research can occur in a number of settings, including public and private hospitals and clinics, other institutions or organisations, community settings, and general or specialist medical practices’ [31].

Emerging drugs were defined as NPS appearing on the market, novel drug classes that are potentially harmful or new formulations of or older drug classes for which problems related to use are emerging.

The survey included a structured process of creating clinical research questions based on the ‘PICOT’ format of: population/patient, intervention, control/comparison, outcome and time [32]. Respondents were provided with two examples followed by six tables providing opportunities to enter PICOT questions with free text; three tables related to methamphetamine and three related to emerging drugs of concern. Survey respondents were also informed at the outset that the PICOT format could be skipped, and a free text box was available for completion further down the page. No logic formulas were included to ‘force’ a response to any of the survey questions. Participants were given two options for free‐text responses, designed to be intentionally open‐ended. The first asked ‘what other comments or research areas with relation to the treatment of substance use disorder due to methamphetamine or emerging drugs do you feel are important?’ and the second asked ‘do you have any other comments or suggestions that may help to determine clinical research priorities in the sector?’. This aimed to provide an alternative format to improve accessibility to non‐researchers and/or non‐clinicians. The type of research questions (e.g. intervention, diagnosis, health service, etc.) was intentionally not defined to allow participants to drive the agenda. Demographic information collected from responders included type of stakeholder, area of expertise, profession, state of residence and gender. The survey was anonymous. Participants were provided with contact details separate from the survey if they wanted to receive information about results or clinical research arising from the survey.

NCCRED staff (including authors KJS, NE) conducted a thematic analysis of survey responses and prepared a report outlining key themes and differing responses between stakeholders. Emergent themes were assessed against published literature through a rapid search of peer‐reviewed databases (to identify if responses were reflective of evidence gaps). All results (source data, thematic analyses, rapid literature review) were presented to the expert panel, which was convened following both an open‐call for expressions of interest and a targeted recruitment effort to ensure an appropriate mix of expertise, skills, consumers and priority populations. The expert panel consisted of 12 stakeholders comprising: a person with lived experience, a person who identified as Aboriginal, a psychologist, medical directors of alcohol and other drugs (AOD) services (inpatient and outpatient, two also holding academic appointments), a senior staff specialist (AOD clinician), a peer‐organisation member, a clinical research coordinator and a clinical toxicologist, all of whom volunteered their time. Author RA, who has had experience in setting research and public health agendas previously, oversaw the panel. Authors KJS, NE and NCCRED project and administrative staff were available to answer questions at the panel review as to earlier phases of the study and to act as secretariat in preparation for, at and in follow up to the meeting.

The expert panel convened to review the survey results, thematic analyses and rapid literature review; to undertake a priority setting recommendation exercise, facilitated by author RA. This included ranking themes identified in the data against a pre‐established set of criteria (current knowledge, answerability, effectiveness, deliverability, burden of disease, equity, novelty, potential for translation, affordability/feasibility, acceptability/ethical aspects, applicability and rationale). During the full‐day workshop, via discussion and consensus review of responses and panel expertise, priorities were further refined and transcribed into a table. Panel members, over the following 3 weeks, selected three priorities in rank order and submitted to the Chair (author RA). Results were tallied across all panel members to determine the key priorities.

This report satisfies the REPRISE guidelines for reporting on priority setting with stakeholders [23], which was published after the completion of our study, and identifies 31 reporting items to include when reporting on priority setting of health research. The completed checklist is available in Table S1 (Supporting Information).

All authors contributed to the study design, development and/or reporting.

## Results

The survey was available and promoted for 4 weeks (7 February–7 March 2019). The survey received 47 individual responses (average rate of completion of questions once the survey was started was 90%, average time spent completing the survey was 12 min). Survey respondents' mean age was 42 years (SD 13 years) and 49% (*n* = 23) identified as female, 45% (*n* = 22) as male and 5% (*n* = 2) as other/preferred not to say. Respondents were predominantly researchers (53%, *n* = 25) and/or clinicians (45%, *n* = 21). Family/friend/caregiver of someone who uses/used methamphetamine or emerging drugs of concern was identified by 15% (*n* = 7) of respondents, and someone who uses/has used methamphetamines or emerging drugs of concern was identified by 13% (*n* = 6) of respondents. For respondents working in the AOD sector, the average length of time working in the sector was 12.2 years (SD 7.4 years). Participants were from all Australian states and territories except Tasmania and the Northern Territory.

The brief literature review outlined current reports on psychosocial therapies and pharmacotherapies for methamphetamine dependence and pill/drug testing, based on the highest volume of common responses to the survey.

Key themes and priorities identified by the expert panel are presented in Table 3. Following the full‐day workshop, each panel member provided their top ranking priorities, which were tallied to determine the top three highest ranking priorities. For methamphetamine clinical research these were: (i) overcoming barriers to intervention uptake (e.g. at time of crisis in emergency departments or primary health care); (ii) pilot pharmacotherapy trials for adults seeking treatment; and (iii) effective communication strategies to consumers on available treatments and the evidence‐based options. For emerging drugs of concern the top priorities were: (i) fixed‐site community located drug checking/‘pill testing’ (connected to an early warning system); (ii) feasibility of social media and other creative opportunities to alert consumers and reduce harm; (iii) GHB overdose and withdrawal management (ranked equally for third highest scoring priority); and (iv) early warning system/shared information system (pooling and sharing of information—with consumers and amongst stakeholders) and its impact on reducing harm (ranked equally for third highest scoring priority). The results of the priority setting process have been presented at scientific meetings, to the Department of Health Australia, and to the public via NCCRED's social media and website.

**Table 3:** Identified themes and priorities—prior to ranking

Theme	Priority
Methamphetamine clinical research	
Adults seeking treatment intervention/treatment research: psychosocial	Contingency management
	Community reinforcement approach
	Cognitive training to provide structured interventions
	Brief intervention (emergency departments)
	Family/network engagement
Adults seeking treatment intervention/treatment research: pharmacotherapy	Methamphetamine withdrawal
	Pilot pharmacotherapy studies
Special populations	Culturally adapted interventions for Indigenous people
	Young person specific interventions
	Clinical interventions for cognitive impairment secondary to methamphetamine use
Health systems/services research	Develop and describe models of care
	Overcoming barriers to intervention uptake (e.g. at time of crisis in emergency departments or primary health care)
	Develop methods for engaging families to assist the treatment journey
	Best practice methods for engaging non‐treatment seeking adults
	Effectiveness of behaviour change communication for consumers
	Messaging and communication to families and concerned others about resources and options
	Effective communication strategies to consumers on available treatments and the evidence‐based options
	Improving research or evidence‐aware culture amongst the clinical workforce
	Health sector workforce behavioural change, knowledge translation (e.g. testing leadership/change agent models)
	Data linkage studies
Other	Feasibility of social media and other creative opportunities as an early warning system for consumers
	Best practice in withdrawal management
Emerging drugs clinical research	
Evidence for effectiveness of drug checking	Fixed‐site community located drug checking/pill testing (connected to an early warning system)
Intervention/treatment research	GHB withdrawal management
	GHB overdose prevention and response
	Treatment of psychostimulant related hyperthermia
	Pharmacogenomics study to identify potential genetic risk factors for stimulant related hyperthermia
Health systems/services research	Early warning system/shared information system—pooling and sharing of information impact on reducing harm
	Feasibility of social media and other creative opportunities to alert consumers and reduce harm
	Mechanisms and effectiveness of messaging and communication to health‐care providers that connects early warning systems with harm reduction
	Evidence‐based models of care for new drug threats (e.g. super agonists, such as fentanyl and analogues)
Systematic reviews of evidence	Drug checking/pill testing
	GHB (treatment)
	Risks and harms from pregabalin use

GHB, gamma hydroxybutyrate.

Secondary outcomes to compare and contrast the priorities identified by various stakeholder groups were not feasible due to the low number of survey responses and missing key stakeholder groups (e.g. policymakers, funders and law enforcement). However, one key difference between responses provided by consumers/caregivers as compared to other respondents was a focus on changing drug law reform.

## Discussion

We conducted a four‐phase study to determine clinical research priorities for the management or treatment of methamphetamines and emerging drugs of concern, eliciting key priorities in both areas. For methamphetamine, the priorities were: overcoming barriers to intervention uptake; pilot pharmacotherapy trials; and effective communication strategies for consumers on evidence‐based treatments. For emerging drugs of concern, the priorities were: trials of fixed site drug‐checking/’pill‐testing’; feasibility of social media and other opportunities to alert consumers to reduce harm; GHB overdose and withdrawal; and development of an early warning/shared information system (for consumers and other stakeholders). Our work demonstrates the feasibility of stakeholder collaboration and engagement, including consumers and others with lived experience, in determining clinical research priorities.

To our knowledge, this is the first national clinical research priority setting study in the Australian AOD sector, and the first to be published. Research priority setting studies have been published elsewhere in the AOD sector. One international priority setting study examined tobacco control and smoking cessation, with 304 stakeholders from 28 countries identifying 183 research questions [33]. Similar to our study, the authors used published processes as guidance, but adapted these to suit their purposes. The study involved a broad range of stakeholders, including consumers, surveying stakeholders to identify specific research questions in first a broad survey, then a ranking process [33]. On a national level, one editorial report of a Chinese addiction research priority setting study outlines a process that limited involvement to only researchers [34]. That report identified 30 key topics (the highest ranking being smoking and alcohol); however, the number of stakeholders consulted was unclear [34]. Given the relatively small population of Australia, the number of stakeholders engaged in our process (47 survey respondents and 12 expert panel members) is not insignificant. An important objective was to include a broad range of stakeholders, particularly given different groups (e.g. clinicians, researchers) are likely to have diverging values or viewpoints. Inclusion of consumers and caregivers was imperative, as research end‐users and those who benefit from research are able to provide a unique and valuable perspective when setting the research agenda [35]. Our study involved consumers at each stage (i.e. by survey and in the expert panel), indicating feasibility of consumer involvement in setting research agendas.

One of the key priorities for emerging drugs was drug‐checking/‘pill testing’. It should be noted that our survey release coincided with the New South Wales Government's *Special Commission of Inquiry into crystal methamphetamine and amphetamine‐type stimulants*, following the death of six young adults at music festivals between December 2017 and January 2019 [36], and public discussions around the planned coroner's inquest into the same music festival deaths [37]. Media and governmental debate regarding ‘pill testing’ was topical and often emotive. This may have influenced our survey findings. As with any priority setting project, these results should be viewed as an ongoing process that should be repeated to reflect contemporary issues.

NCCRED conducted this study in tandem with a systematic review examining pharmacotherapy options for methamphetamine use disorder [38] and a review of Australian clinical guidelines for methamphetamine use disorder [39]. We aimed to evaluate the strength of available evidence and identify gaps in the knowledge base and incorporate these along with the present study findings into NCCRED's strategic direction; to drive NCCRED's programmatic outputs for a 2‐year period (2019–2021). The NCCRED clinical research seed funding, clinical research fellowship and scholarship programs have focused on the identified priorities. To date, interim results of seed funding projects have been presented at the National NCCRED Clinical Research Symposium (Hobart, 2019; online, 2020). Presentations are publicly available on the NCCRED website, as are seed funding reports and outputs associated with funding and fellowship programs [40]. As a result of the NCCRED clinical research seed funding and clinical research fellowship programs, unique partnerships have evolved, such as in the Emerging Drugs Network of Australia project, now funded by a National Health and Medical Research Council competitive grant. Clinical research projects progressed by the Centre also focus on these priorities. Current NCCRED work includes a study of GHB overdose, and a pilot study of lisdexamfetamine for the treatment of acute methamphetamine withdrawal [41].

Based on published methods, a structured, inclusive and transparent process was undertaken that began with broad stakeholder engagement and then a targeted expert panel of diverse skill mix. Various methods of research priority setting have distinct advantages and disadvantages. In some cases, disadvantages include lengthy, resource‐intensive procedures. Selection of a priority setting process therefore requires careful account of the resources available. In a review of 165 health research prioritisation studies (2001–2014), the majority reported using the Child Health and Nutrition Research Initiative method (26%) or the Delphi method (24%). Other methods included James Lind Alliance (8%), Combined Approach Matrix (2%) and Essential National Health Research (<1%). In 19% of studies non‐specific methods were used, such as expert panel interviews and focus group discussions [10]. An additional 8% used an online questionnaire and 8.5% combined a questionnaire with literature review. Of the reviewed publications, 3% did not identify or describe their approach [10]. In that review, only 15 studies were conducted in Australia and, of those, none were in the AOD sector [10].

In one survey of 66 groups in the Cochrane collaboration, under half (43%) had a system in place to inform the prioritisation of topics for Cochrane reviews, and while most groups who reported on a system engaged stakeholders, including researchers, practitioners and patients in their prioritisation, disparate approaches were used [42].

This study has limitations. Presenting the survey with a PICOT format may have created a limitation that prevented non‐research or non‐clinical staff from participating. As with any study that requests stakeholders to share views, there is a risk participants were not forthcoming with ideas due to intellectual property concerns. We attempted to mitigate for these by requesting free‐text or broad commentary in addition to specific research questions. Our study was conducted in Australia; and therefore results may not be generalisable to other jurisdictions.

In our study, although it was our intention, law enforcement, primary research funders and policymakers did not participate. It is possible results would be different had those stakeholders participated. This represents a missed opportunity when setting out to drive the national research agenda. We would recommend future studies involve research funders and policymakers from the outset, as their involvement is imperative for translating the findings of such a study into implementation of funding activities. However, NCCRED commissioned and implemented this study, and given that NCCRED's programmatic outputs include seed and other funding opportunities (fellowships, scholarships), the study was undertaken by a secondary research funder and results were directly implemented into programmatic decisions.

## Conclusion

We demonstrated the feasibility of undertaking a national clinical research priority setting exercise related to methamphetamine and emerging drugs of concern, which identified several priority areas and questions for clinical research in the Australian AOD sector. Using a collaborative approach, stakeholders were engaged in all aspects of the project, maximising the likelihood that future research findings to emerge from this process will be translated to clinical practice and align with consumer experience. The priorities identified here informed the direction of NCCRED. In reporting these findings, we hope these valuable insights, that reflect community concerns, will be taken into account when developing future research priorities in the Australian AOD sector.

## Conflicts of Interest

KJS is an employee of UNSW, NCCRED and has no other interests to declare. NE is an employee of St Vincent's Hospital Sydney and seconded to UNSW, NCCRED and has no other interests to declare. MC is an employee of the Government of Western Australia Mental Health Commission and had no other interests to declare. PH is an employee of the Sydney Local Health District and has no other interests to declare. RA has no relevant interests to declare. This study was funded by NCCRED (funded by the Australian Commonwealth Department of Health).

## Supporting information


**Table S1.** REPRISE reporting checklist.
