# Supplementary material for: A clinical research priority setting study for issues related to the use of methamphetamine and emerging drugs of concern in Australia
Source: Drug Alcohol Rev. 2021 Jul 8;41(2):309–19. doi: 10.1111/dar.13350 (PMC9290984; doi:10.1111/dar.13350)
Supplement: Supplementary file 1 — Table S1. REPRISE reporting checklist. [file DAR-41-309-s001.pdf]

Table S1. REPRISE reporting checklist

REPRISE Checklist and explanatory notes [20]			
A	Context and scope		Manuscript reference
1	Define geographical scope	Global, regional, national, city, local area, institutional/organisational level, health service	Methods, paragraph 3
2	Define health area, field, focus	Disease or condition specific, interventions, healthcare delivery, health system	Methods, paragraph 5
3	Define the intended beneficiaries	This may include the general population or a specific population based on demographic (age, gender), clinical (disease, condition) or other characteristics who may benefit from the research	Introduction, paragraphs 3 & 5; Table 2
4	Define the target audience of the priorities	Policy makers, funders, researchers, industry or others who have the potential to implement the priorities identified	Introduction, paragraph 3; Table 2
5	Identify the research area	Public health, health services research, clinical research, basic science	Introduction, paragraph 6; Methods, paragraph 5
6	Identify the type of research questions	Etiology, diagnosis, prevention, treatment (interventions), prognosis, health services, psychosocial, behavioral and social science, economic evaluation, implementation; this may not be pre-defined	Methods, paragraph 7
7	Define the time frame	Interim, short-term, long-term priorities, plans to revise and update	Table 2
B	Governance and team		
8	Describe the selection and structure of the leadership and management team	Those responsible for initiating, developing, and guiding the process for priority setting, and examples of structures include; Steering Committee, Advisory Group, Technical Experts	Methods, paragraph 8
9	Describe the characteristics of the team	Stakeholder group or role, institutional affiliations, country or region, demographics (e.g. age sex), discipline, experience, expertise	Methods, paragraph 8
10	Describe any training or experience relevant to conducting priority setting	Consultants or advisors, members with experience or skills relevant to the conducting priority-setting e.g. qualitative methods, surveys, facilitation	Methods, paragraph 8
C	Framework for priority setting		
11	State the framework used (if any)	James Lind Alliance, COHRED, CHNRI, Dialogue Model, no framework (general research priority setting)	Methods, paragraph 1; Table 2

For Peer Review Only

D	Stakeholders or participants		
12	Define the inclusion criteria for stakeholders involved in priority-setting	Patients, caregivers, general community, health professionals, researchers, policy makers, non-governmental organisations, government, industry; specific groups including vulnerable and marginalised populations	Methods, paragraphs 3 & 8
13	State the strategy or method for identifying and engaging stakeholders	Partnership with organisations, social media, recruitment through hospitals	Methods, paragraphs 3 & 8
14	Indicate the number of participants and/or organisations involved	Number of individuals and organisations, include number by stakeholder group	Methods, paragraph 7; Results, paragraph 1
15	Describe the characteristics of stakeholders	Stakeholder group, demographic characteristics, areas of interest and expertise, discipline, affiliations	Methods, paragraph 8; Results, paragraph 1
16	State if reimbursement for participation was provided	Cash, vouchers, certificates, acknowledgement; what purpose e.g. travel, accommodation, honorarium	Methods, paragraphs 5 & 8
E	Identification and collection of research priorities		
17	Describe methods for collecting initial priorities	Methods e.g. Delphi survey, surveys, nominal group technique, interviews, focus groups, meetings, workshops; prioritisation e.g. voting, ranking; mode e.g. face-to-face, online; may be informed by evidence e.g. systematic reviews, reviews of guidelines/other documents, health technology assessment	Methods; Table 2
18	Describe methods for collating and categorising priorities	Taxonomy or other framework used to organise, summarise, and aggregate topics or questions	Methods; Results paragraph 4; Table 2
19	Describe methods and reasons for modifying (removing, adding, reframing) priorities	Based on scope, clarity, definition, duplication, other criteria	Table 2
20	Describe methods for refining or translating priorities into research topics or questions	Reviewed by Steering Committee or project team	Results paragraph 3; Table 2
21	Describe methods for checking whether research questions or topics have been answered	Systematic reviews, evidence mapping, consultation with experts	Methods, paragraph 8; Discussion, paragraph 4

For Peer Review Only

22	Describe number of research questions or topics	Number of priorities at each stage of the process	Results, Table 3
F	Prioritisation of research topics/questions		
23	Describe methods and criteria for prioritising research topics or questions	Methods e.g. Delphi survey, surveys, nominal group technique, interviews, focus groups, meetings, workshops; Prioritisation e.g. voting, ranking; Mode e.g. face-to-face, online; Criteria e.g. need, feasibility, novelty, equity	Table 2
24	State the method or threshold for excluding research topics/questions	Thresholds for ranking scores, proportions, votes; other criteria	Results paragraphs 4 &5; Table 2
G	Output		
25	State the approach to formulating the research priorities	Area, topic, questions, PICO (population, intervention, comparator, outcome)	Methods paragraph 7; Table 2
H	Evaluation and feedback		
26	Describe how the process of prioritisation was evaluated	Survey, workshop	Remains on the agenda
27	Describe how priorities were fed back to stakeholders and/or to the public; and how feedback (if received) was addressed and integrated	Public meetings or workshop, newsletters, website, email, online presentations	Results, paragraph 3
I	Implementation		
28	Outline the strategy or action plans for implementing priorities	Communication with target audience, via policies and funding	Discussion, paragraph 4
29	Describe plans, strategies, or suggestions to evaluate impact	Integration in decision-making, funding allocation, review of relevant documents	Discussion, paragraph 4
J	Funding and conflict of interest		
30	State sources of funding	Name sources of funding for the priority-setting exercise; if relevant include the budget and/or cost	Manuscript Conflicts of Interest

For Peer Review Only

31	Declare any conflicts or competing interests	State any conflicts of interest that may be at an individual level and/or at a contextual level (e.g. political issues, controversies) that may affect the process, output or implementation.	Manuscript Conflicts of Interest

CHNRI, Child Health and Nutrition Research Initiative; COHRED, Council on Health Research for Development.

For Peer Review Only
